# Supplementary material for: Revised historical Northern Hemisphere black carbon emissions based on inverse modeling of ice core records
Source: Nat Commun. 2023 Jan 17;14:271. doi: 10.1038/s41467-022-35660-0 (PMC9845213; doi:10.1038/s41467-022-35660-0)
Supplement: Supplementary file 1 — Supplementary Information [file 41467_2022_35660_MOESM1_ESM.pdf]

# Revised historical Northern Hemisphere black carbon emissions based on inverse modeling of ice core records

Sabine Eckhardt[1*], Ignacio Pisso[1], Nikolaos Evangeliou[1], Christine Groot Zwaaftink[1], Andreas Plach[2], Joseph R. McConnell[3], Michael Sigl[4,5] Meri Ruppel[6,7], Christian Zdanowicz[8], Saehee Lim[9], Nathan Chellman[3], Thomas Opel[10], Hanno Meyer[10], Jørgen Peder Steffensen[11], Margit Schwikowski[12] and Andreas Stohl[2]

## Supplementary Information

### Details for the ice-core BC observations/locations

| Name (abbreviation) | Coordinates | elevation (a.s.l.) | CERA topography | Precipitation in CERA | Measurement method | Reference |
|---|---|---|---|---|---|---|
| **Flade Isblink (FLI)** | 81.6°N, 15.7°W | 618 m | 249 m | 272.3 mm | Continuous SP2 (DRI) | This study |
| **Akademii Nauk (AN)** | 80.5°N, 94.8°E | 750 m | 56 m | 230.5 mm | Continuous SP2 (DRI) | This study, Opel et al. 2013 |
| **Holtedahlfonna (HOL)** | 79.1°N, 13.3°E | 1150 m | 312 m | 324.6 mm | Thermo-optical | Ruppel et al. 2014 |
| **Lomonosovfonna (LOM)** | 78◦49′N, 17◦25′E | 1202 m | 499 m | 391.9 mm | Discrete SP2 (PSI) | Osmont et al., 2018 |
| **Humboldt (HUMB)** | 78.3° N,56.5°E | 1985 m | 2000 m | 137.2 mm | Continuous SP2 (DRI) | Bauer et al., 2013; Sigl et al., 2013 |
| **Tunu** | 78°N, 33.9°W | 2000 m | 2185 m | 76.3 mm | Continuous SP2 (DRI) | Grieman et al., 2018 |
| **North Greenland Eemian (NEEM)** | 77.5°N, 51.5°W | 2480 m | 2347 m | 116.7 mm | Continuous SP2 (DRI) | Sigl et al., 2013 ; Zennaro et al., 2014 |
| **Devon (DEV)** | 75.3°N, 81.6°W | 1903 m | 431 m | 220.2 mm | Continuous SP2 (Curtin Univ., Australia) | Zdanowicz et al., 2018 |
| **Summit (SUM)** | 72.6°N, 37.6°W | 3200 m | 3177 m | 159.9 mm | Continuous SP2 (DRI) | Keegan et al., 2014 |
| **D4** | 71.4°N, 43.9°W | 2766 m | 2728 m | 276.2 mm | Continuous SP2 (DRI) | McConnell et al., 2007 |
| **ACT2** | 66°N, 43.6°W | 2410 m | 2461 m | 640.8 mm | Continuous SP2 (DRI) | McConnell and Edwards. 2008 |

| | | | | | | |
|---|---|---|---|---|---|---|
| **Colle Gnifetti (CG)** | 45.9° N,7.9°E | 4450 m | 1166 m | 1315.4 mm | Discrete SP2 (PSI) | Sigl et al., 2018 |
| **Mt. Elbrus (ELB)** | 43.3°N, 42.5°E | 5115 m | 1349 m | 1234.6 mm | Continuous SP2 (IGE) | Lim et al., 2017 |

**Table S1:** Names, locations, heights, corresponding CERA topographical height and CERA annual mean precipitation, measurement method and available references for the 13 ice cores used in this study.

**Emissions**

For transport into the Arctic, high- and mid-latitude emissions are more relevant than global emissions. Therefore, in Fig. S1 we present time series of the BC emissions north of 30°N, separately for North America, Europe, Russia, and South Asia, as well as for all regions. When considering all regions together, the biomass burning emissions, even though showing inter annual variability, remained rather constant over the last hundred years, whereas the anthropogenic emissions increased strongly until 1980s, with a sharp decrease in the last decade of the period in the CMIP6 data set but not as steep in the CMIP5 data set. At the beginning of the time series, anthropogenic BC emissions were lowest at 0.6 and 0.9 Gt/year for the CMIP5 and CMIP6 data sets, respectively. There is a much steeper increase in the CMIP5 emissions in the 19$^{th}$ century, followed by a peak of 2.6 Gt/year in the year 1920, a leveling out until 1970 and another steep increase in the 1970s. The CMIP5 maximum in 1920 is mostly due to emissions in North America peaking at that time, while the increase from the 1970s is mostly due to Russian and Asian emissions. However, Russian emissions collapsed in the 1980s, leading to relatively constant global emissions north of 30°N in the 1980s. The CMIP6 emissions show a first peak already in 1915; however, this peak is much less pronounced than the 1920 peak in the CMIP5 dataset, which is caused by generally much lower CMIP6 than CMIP5 emissions in North America. The total CMIP6 emissions then stayed relatively constant until about the year 1950. After that, CMIP6 emissions peak first in Europe, then Russia and finally Asia, which caused strongly increasing total emissions until about 1980, after which emissions decreased in the final decade. The differences between CMIP5 and CMIP6 anthropogenic emissions are remarkably large, both in terms of total magnitude, temporal evolution and regional contributions. While in Europe anthropogenic emissions are dominant throughout the whole period, in North America and Russia BB emissions contribute a substantial fraction of the total emissions, especially at the beginning and at the end of the time series when anthropogenic emissions were relatively low. In Russia, BB emissions contribute up to 62% (CMIP5) and 74% (CMIP6) of the total emissions and in the last decade around one quarter for both inventories.

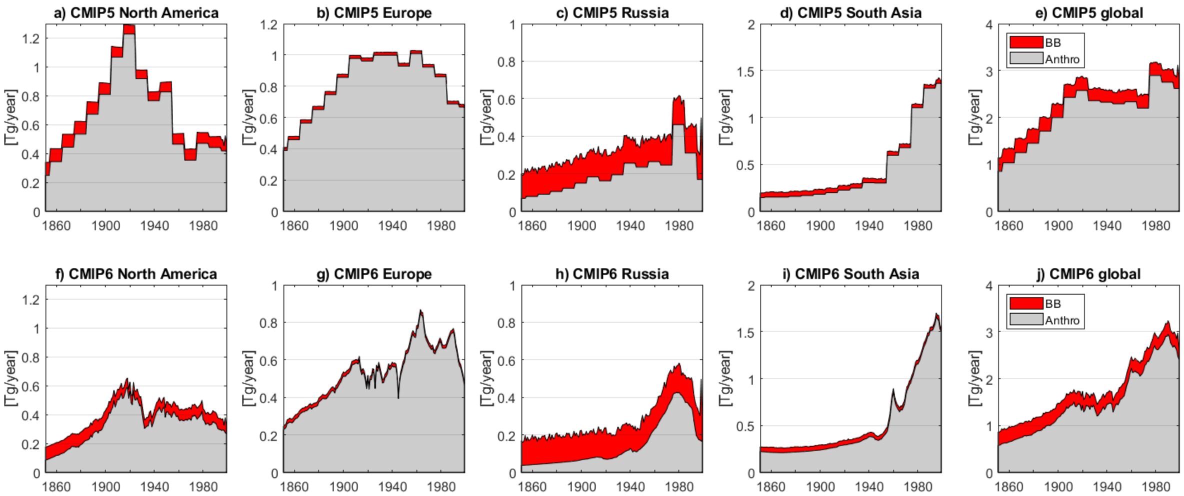


**Fig S1: Annual anthropogenic (grey) and BB (red) BC emissions for the period 1850 to 1999. All panels show only emissions north of 30°N for North America (a, f), Europe (b, g), Russia (c, h), Southern Asia (d, i) and all regions together (e, j). The regions used are depicted in Fig. 5. Both upper and lower panels show identical BB emissions (van Marle et al., 2017), but the upper panels (a-e) show CMIP5 and the lower panels (f-j) CMIP6 anthropogenic emissions.**

## Variation of BC emissions related to measured and modeled BC deposition in ice cores

For comparing the magnitude of modeled and observed BC deposition fluxes, we focus on the last 10 years (1990-1999) of our record when emission uncertainties are expected to be smallest. Fig. S2 shows average 1990-1999 observed BC deposition fluxes, compared to modeled values using both the CMIP5 and CMIP6 emission inventories. On average the modeled deposition fluxes based on CMIP5 and CMIP6 are a factor of 3 higher than the observations. For the Greenland locations the highest ratio is 3.7 for Summit. NEEM, Flade Isblink and Akademii Nauk fit best with a ratio of 0.9 to 1.3. The highest underestimation is found for the Holtedahlfonna ice core at Spitsbergen with a factor of 0.3. Colle Gnifetti is overestimated by 4.4 and Mt Elbrus observations are showing a factor of 1.2. The overestimation in the Alps might occur because of Colle Gnifetti is very special in that it is located on a wind exposed small glacier saddle. Unconsolidated light winter snow is therefore not preserved at the site and the seasonal distribution is heavily biased towards the summer season (when only a small fraction of the anthropogenic BC emissions occurred).

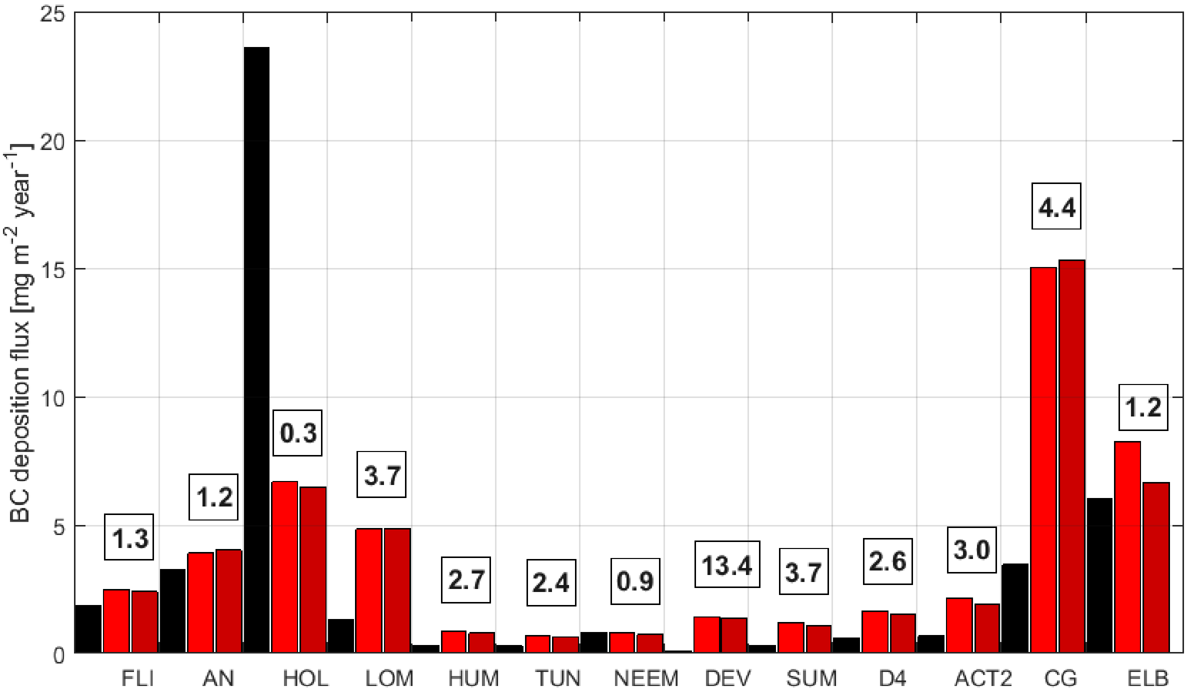


**Fig S2: Average measured (black) and modeled (based on CMIP5 light red, based on CMIP6 dark red) BC flux for the years 1990-2000. The labels show the ratio between the average modeled (average of CMIP5 and CMIP6) and the measured deposition flux. (Abbreviations are explained in Table S1)**

In general, the model overestimates the deposition at all sites where BC was measured with SP2 instruments. This may partly result from the SP2 measurements generally presenting underestimations of actual BC concentrations, for instance, due the limited particles size range detected, limitation of size distribution obtained by SP2 and calibration uncertainties (e.g. Schwarz et al., 2012; Schwarz et al., 2013; Wendl et al., 2014; Kaspari et al., 2014; Lim et al., 2014). In addition the wind scouring that removes part of the wintertime BC deposition, could also lead to lower values at Devon and Colle Gnifetti. Only at Holtedahlfonna, where BC measurements were made with the thermal-optical method, strong model underestimates were found, NEEM has slight underestimation. Given that there are substantial systematic differences of about a factor 3 between the different measurement methods (Sharma et al., 2017; Lim et al., 2014; Osmont et al., 2018), it is likely that the biases between the observations and the modeled data at Holtedahlfonna are real or mostly attributable to ambiguities in the measurement of BC between the thermal-optical method and model, and to a lesser extent possible true underestimation of the model. In addition to modeling biases, systematic differences may also originate from inaccurate emission data, since emission factors used to calculate the inventories, also were measured with different instruments.

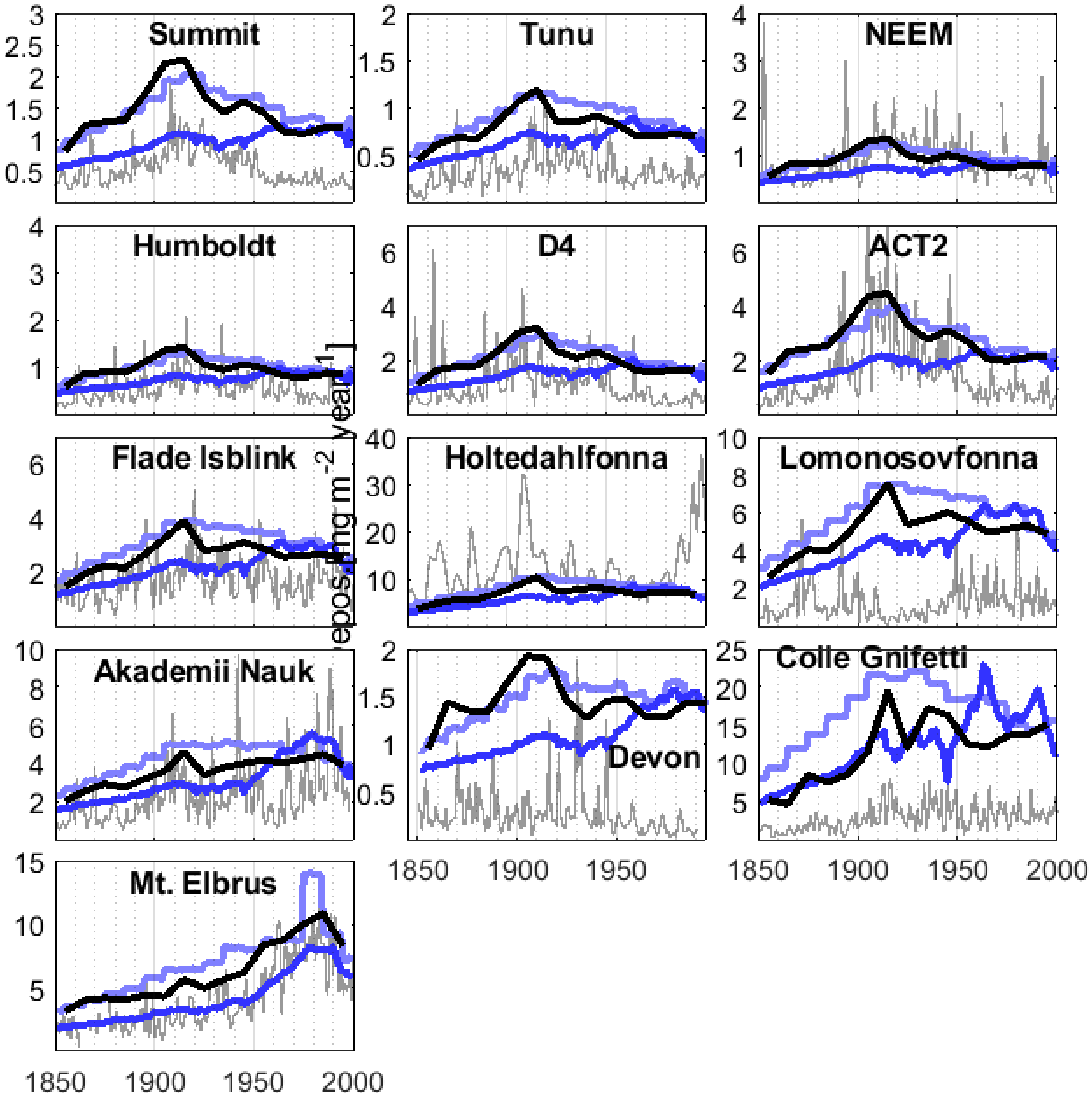


**Fig S3: Same as Fig. 2, but without scaling**

**Source regions for modelled BC depositions**

Figure S4 shows BC emission contributions based on CMIP6 (the newer one of the two inventories) emission data and FLEXPART emission sensitivities combined for wet and dry deposition, for two ice cores with pronouncedly different characteristics: D4 (Fig S4a, b), which has its source region over both North America and western Europe (compare with Fig. 1b) and Akademii Nauk (Fig. S4c, d), which receives emissions mostly from wide parts of Eurasia but has little sensitivity to North American emissions (see Fig. 1c). As both emission magnitude and distribution changed over the 150 years investigated, we plot the emission contributions separately for two ten-year periods (1910-1920 and 1980-1990, respectively). At the beginning of the 20th century (Fig S4a), there are two source contribution hot spots for D4, one over the North American East coast and one over western

Europe (primarily Great Britain). At the end of the 20th century (Fig. S4b), the source contributions from both these regions are reduced substantially, but additional contributions come from emissions in Eastern Europe and Northern Siberia (gas flaring), where the emission sensitivity is quite low. This eastward shift of emission contributions over Eurasia is even more pronounced for Akademii Nauk. There, source contributions at the beginning of the 20th century are mainly from western Europe (Fig. S4c), but at the end of the 20th century (Fig. S4d), substantial source contributions occur also in eastern Europe, and the gas flaring region in northern Siberia has become the largest source (Stohl et al., 2013).

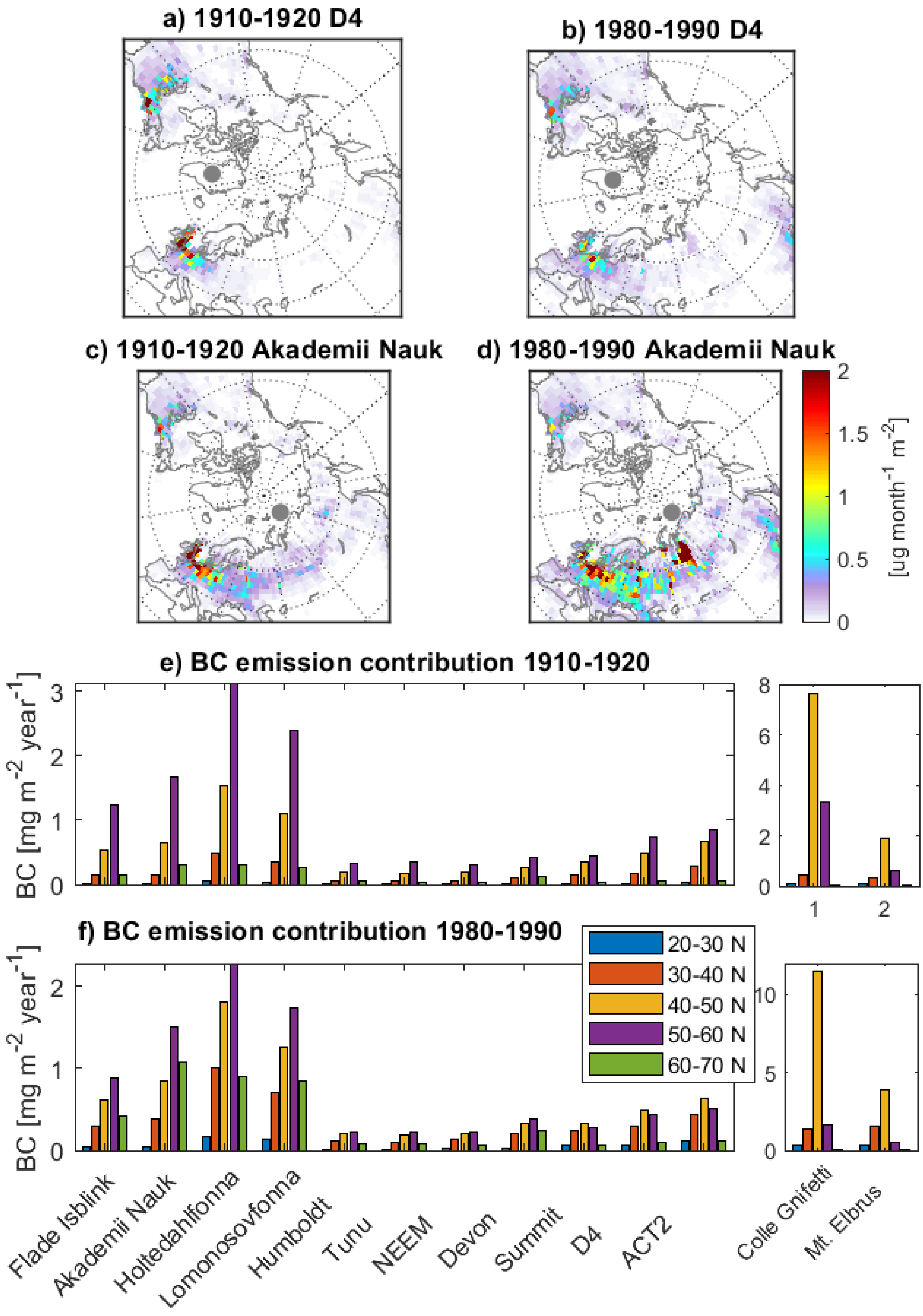


**Fig S4: 10 year averaged monthly emission contribution (μg m$^{-2}$ month$^{-1}$) based on the CMIP6 (BB and anthropogenic) emissions for D4 (panels a, b) and Akademii Nauk (panels c, d) for the period of 1910-1920 (panels a, c) and 1980-1990**

**(panels b, d). The measurement locations are depicted as grey dots. Panels e and f show the emission contributions for all 13 ice cores for 10-degree latitude bands averaged over 1910-1920 (panel e) and 1980-1990 (panel f).**

In order to see the emission contributions from different latitudes on the deposition at the ice core locations, we sum up the contributions for all sites over 10-degree latitude bands in Fig S4 (panels a-d) for the decades 1910-1920 and 1980-1990, respectively. The contributions north of 30ºN are responsible for the majority of all BC deposited at the Arctic ice core locations. Emission contributions from the 60-70°N latitude band show a significant increase because of the high emissions in the gas flaring region in Northern Russia (Stohl et al., 2013).

A time series of simulated BC deposition, split by emission region origin, is created by integrating the source contribution maps for CMIP6 emissions, shown exemplarily for two ice core sites in Fig. S4, over defined emission regions (Fig. S5). We distinguish between North American, European, Russian and Southern Asian and the rest of the world emissions (Fig. S5, last panel). The high-altitude Greenland ice cores receive about equal contributions from Europe and North America. Averaged over the 100 years period, North America contributes between 27% (Tunu) and 36% (NEEM) of the BC, while Europe contributes between 37% (NEEM) and 48% (Tunu). After 1960, additional contributions from Russia and Asia become clearly visible. For lower altitude locations, the source contributions are dominated by one or two of the defined regions. At the low-altitude sites in the western hemisphere (Humboldt), North American emissions are most important, while for Akademii Nauk, Flade Isblink, and Holtedahlfonna, European and Russian emissions dominate. At these sites, a reduction in European contributions and an increase of Russian contributions can be observed during the last few decades of the 20th century. For the last two decades, the Russian contributions are 50% at Akademii Nauk, 31% at Flade Isblink, and 28% at Holtedahlfonna. A substantial part of increased contribution from Russia are a result of gas flaring in high-latitude Siberia, that has likely increased along with increasing oil and gas production in Russia since 1960s (OPEC, 2018), and for instance, in 2010 accounted for ca. 36 % of all BC emissions from Russia (Huang et al., 2015).The alpine site Colle Gnifetti receives its BC depostion almost exclusively from Europe, while BC at Mt. Elbrus originates both from Russia and the Southern Asia, with a smaller contribution from Europe.

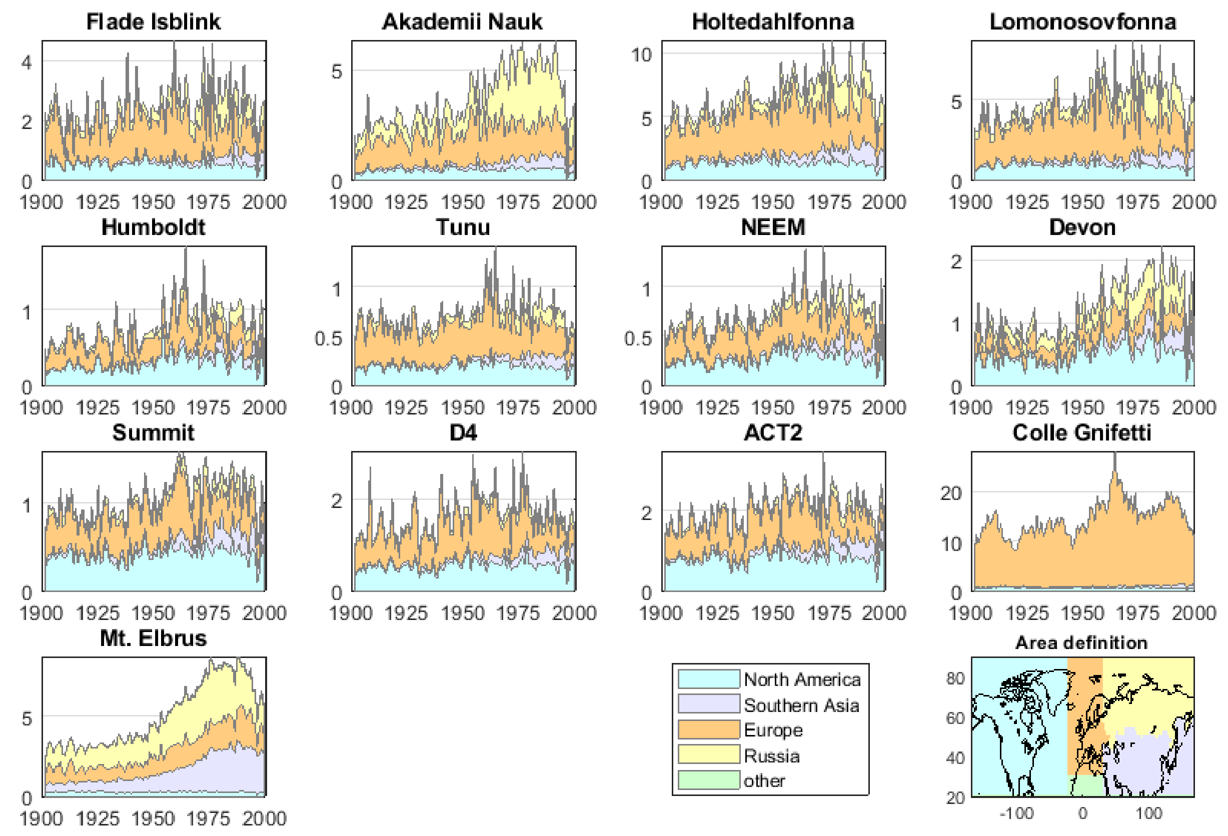


**Fig S5: Annual modeled BC deposition values (mg m$^{-2}$ year$^{-2}$), based on CMIP6 emissions, split by source region (North America - blue, Asia without Russia (South Asia) - magenta, Europe - orange, Russia - yellow–, rest of the world - green) for all investigated ice cores. The last panel shows the definition of emission regions used for the analysis.**

The transport patterns and the source regions for Arctic aerosols vary with location and season. During winter and early spring, fast low-altitude transport of high latitude emissions into the Arctic is possible. In addition to Russian gas flaring emissions, domestic combustion sources, with their maximum during the cold period, are important (Klonecki et al., 2003; Stohl et al., 2013). In summer, the transport into the Arctic occurs mostly at higher altitudes coinciding with an enhanced level of wet scavenging. During this season, BB emissions are frequently transported into the Arctic. Therefore, even though atmospheric surface BC concentrations are at a minimum, large amounts of BC can be deposited in the Arctic in summer, in particular along its fringes. BB emissions from Canadian wildfires have also been identified as an important source of BC deposited on the Greenland ice sheet (McConnell et al., 2007; Thomas et al., 2017). Indeed, the Devon and NEEM ice cores show large contributions from forest fires (35% and 23%, respectively) (Fig. S6, lower panel).

We find that wet deposition dominates at most sites (Fig. S6, upper panel). This is particularly true in winter, when dry deposition is almost completely negligible. The inefficiency of dry deposition in winter is related to the high static stability in the Arctic lower troposphere, which almost completely stops the dry deposition process for small BC particles, which have no significant gravitational settling. Thus, dry deposition in winter contributes less than 1% of total winter deposition. At the same time, winter and spring wet deposition make the largest contributions to the total annual deposition at all Arctic sites (Fig. S6). While summer deposition is generally lower, dry and wet deposition are comparable at all sites during this season.

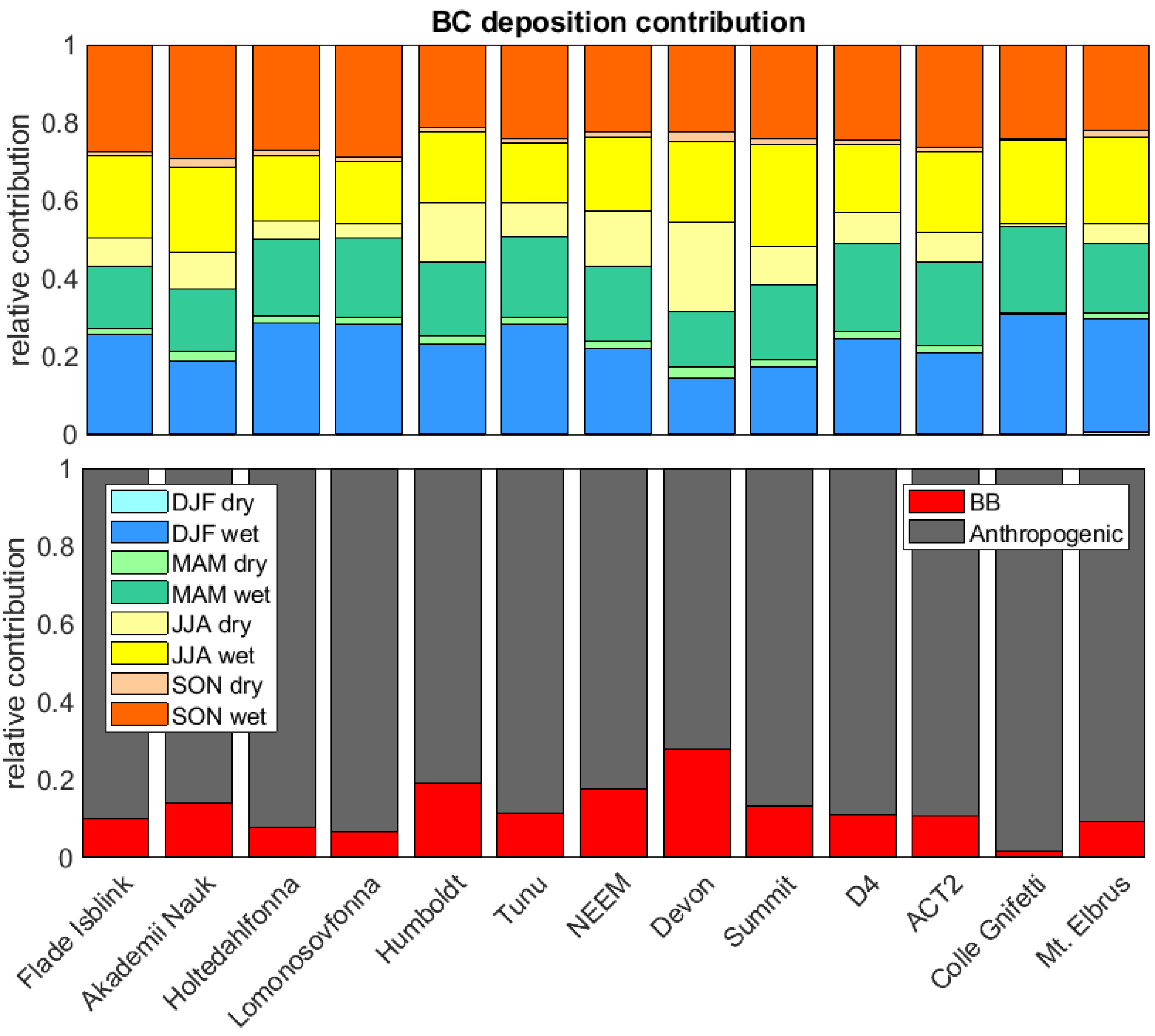


**Fig S6: Relative contributions to BC deposition fluxes modeled with CMIP6 emissions and averaged from 1900-1999 for the different ice core sites. Relative contributions are shown for the four seasons and split into wet and dry deposition (upper panel), and displayed separately for anthropogenic and biomass burning (BB) contributions (lower panel).**

To evaluate the evolution and magnitude of the BC emissions, we plot both the CMIP5 and CMIP6 (1850-1999) emissions, split into different latitude bands, against the ice core observations (Fig. S7 and Fig. S8, respectively). As most ice cores are located in the Arctic, emissions from the higher latitude bands are more important and therefore we stack the emissions starting from the highest to the lowest latitudes with darkest shading for the highest latitude bands. Most of the emission contributions to Arctic ice core deposition are from latitudes north of 30˚N (Fig. S1e, f), and therefore we further emphasize the emissions north of 30˚N with a white line.

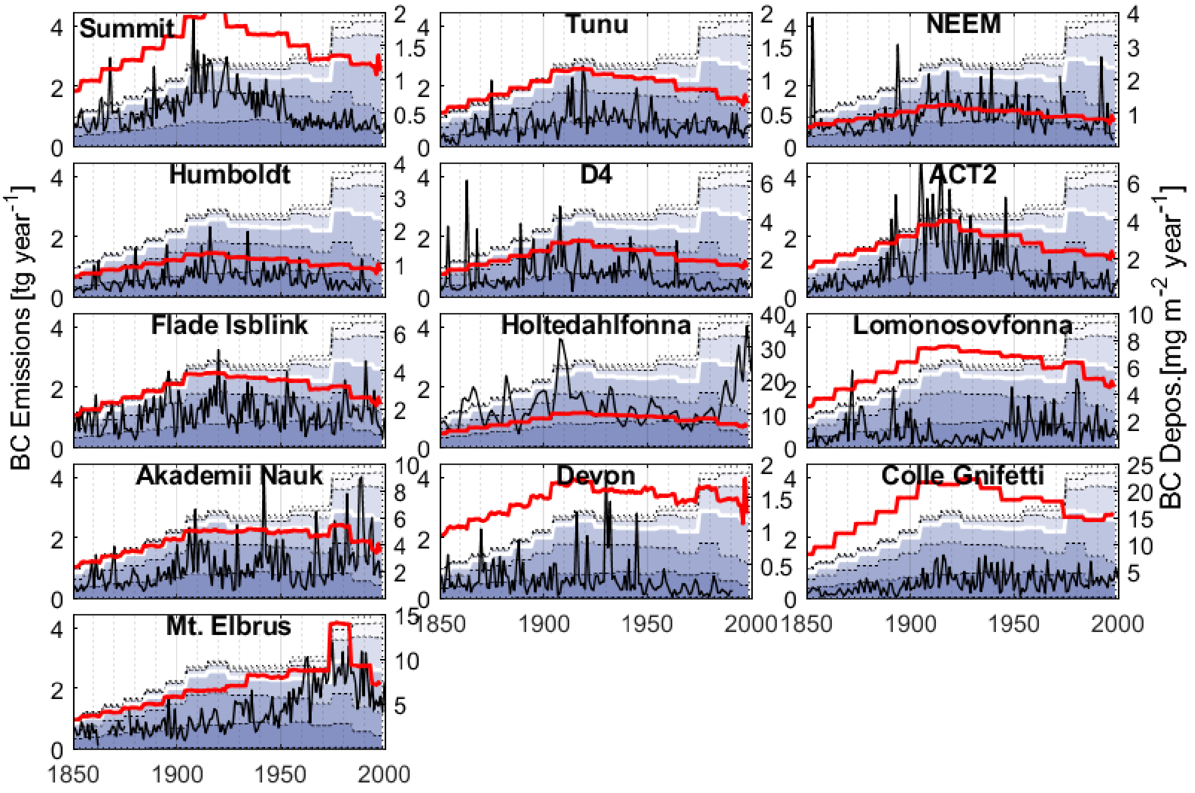


**Fig S7: Time series of BC emissions in the Northern Hemisphere from the CMIP5 inventory (y-axis to the right). Emissions from 10-degree latitude bands are stacked upon each other and shown with different shading, from 0-10°N (white) to 60-70°N (blue). Emissions from 70-90°N are minor and not visible on this scale. The white line shows the total emissions north of 30°N. Superimposed on the emissions are the measured (black) and modeled (red) annual BC deposition values for the different ice core sites (y-axis to the left). The modeled values were obtained by using the BB emissions and the CMIP5 inventory combined with monthly SRRs averaged over the 100-year CERA-20C period. Notice that the ordinate scales for the deposition values are on the right-hand side of each panel and are different for the different ice core sites.**

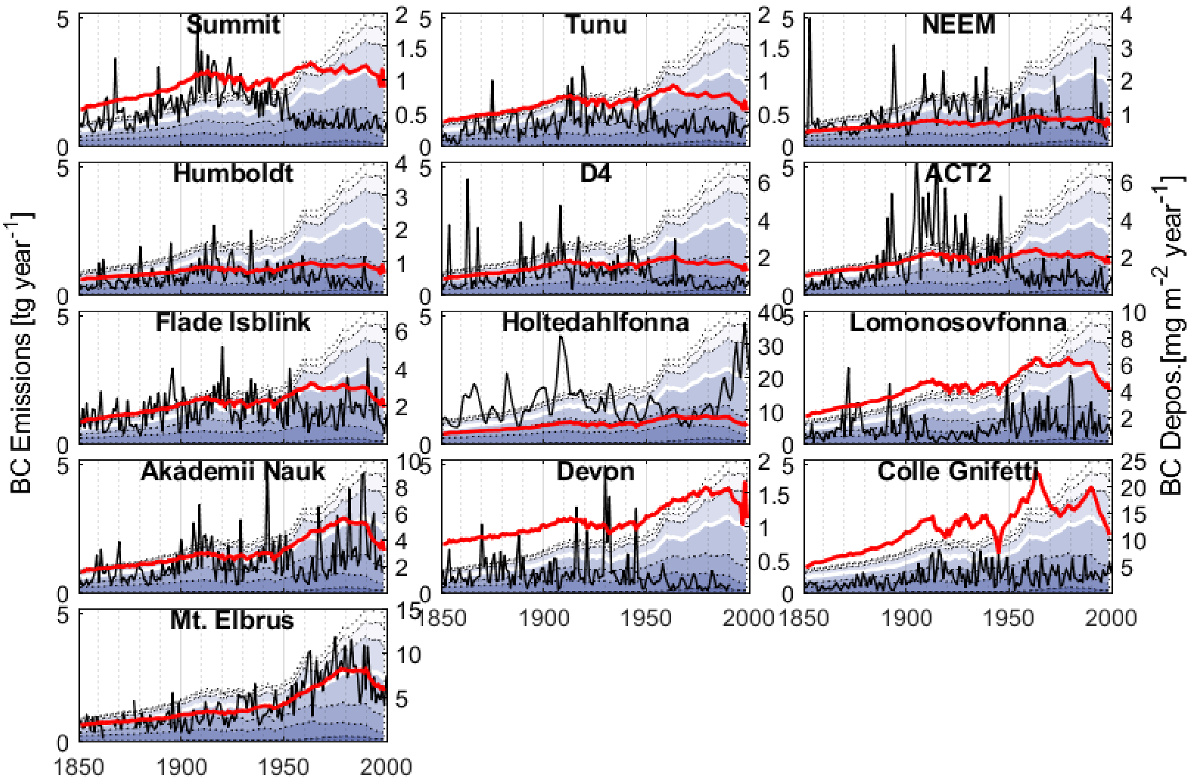


**Fig S8: Same es Fig. S7, but for CMIP6**

Superimposed on the emissions, we show the measured (black line) and modeled BC deposition fluxes for the ice cores in Fig. S7 and S8. The measured mean deposition fluxes span about one order of magnitude, with the lowest values occurring for the high altitude Greenland sites (around 0.5 mg/m$^2$/year) and the highest values occurring at Mt. Elbrus (4.2 mg/m$^2$/year) and Holtedahlfonna 12.3 mg/m$^2$/year, but note that Holtedahlfonna is the only site where a thermo-optical measurement method has been used which is known to produce higher concentrations. This difference is reflected well in the modeled fluxes which span from 0.9 mg/m$^2$/year for Tunu to 7.0 mg/m$^2$/year for Mt. Elbrus in CMIP5 and 0.6 mg/m$^2$/year to 4.1 mg/m$^2$/year for CMIP6.

In several ice cores (ACT2, Summit, Colle Gnifetti, D4, Holtedahlfonna, Akademii Nauk) the measured values peak around the year 1910 and then most show a decrease until the year 1999. At ACT2, D4 and Summit, measured concentrations after 1950 are only about 25% of the highest concentrations at the beginning of the 20th century, without any further prominent increases. The Humboldt ice core shows an almost linear decrease from the early 20th century until today. By contrast, the Akademii Nauk and Holtedahlfonna ice cores show a second peak in 1980, which lead to the highest values in the entire record at Holtedahlfonna in 1998 and a subsequent decrease. Such high late-20$^{th}$ century BC deposition fluxes were also observed in lake sediments from north-western arctic Russia, and were attributed to high emissions in the Russian Arctic flaring region (Ruppel et al., 2021), an important source region for both Akademii Nauk and Holtedahlfonna (Fig. 2).

The timing of the observed early 20$^{th}$ century BC deposition maximum in Greenland ice cores (ACT2, D4, Summit, Tunu, NEEM) is much better captured by the model when using CMIP5 emissions (Fig. S7) than CMIP6

emissions (Fig. S8). These sites are most sensitive to emissions in North America (Fig. 1; S5), and indeed in North America the highest CMIP5 emissions of the whole period occurred already in 1910. Our comparison confirms that there must have been a strong North American BC emission peak already in the early 20th century declining from there onwards. In contrast, the CMIP6 FLEXPART simulations do not capture the observed early 20$^{th}$ century deposition maximum, suggesting that the temporal evolution of BC emissions in North America in the 20th century is not correctly represented in the CMIP6 emission inventory.

The measured and the CMIP6 modeled deposition fluxes at NEEM are similar during the first 50 years, but thereafter the modeled deposition values are higher than the observed ones (Fig. S8). This is not the case at Tunu and Summit, which exhibit much lower measured fluxes and BC deposition at both sites is significantly overestimated by the model throughout the entire record.

In contrast to the Arctic sites, Mt. Elbrus observations show increasing BC deposition until the 1970s, followed by relatively stable values decreasing only in the 1980s. This evolution is consistent with the continuous increase in lower-latitude emissions both in CMIP5 and CMIP6 data (see Fig. S1, panels c and h), which only ended in the 1980s. Especially the emissions over South Asia and partly over Russia have increased after 1970 according to both emission inventories. While the Mt. Elbrus record shows this particularly clearly, it can also be seen at Holtedahlfonna, Akademii Nauk and, less clearly, at Flade Isblink, which are also strongly influenced by Russian emissions. On the other hand, ACT2, Summit, D4 and Tunu have very low deposition fluxes during this period. This strongly suggests that the ca 1980 maximum in the Akademii Nauk and Holtedahlfonna ice core mostly can be attributed to emissions from the former Soviet Union, which dropped in the 1980s after the collapse of the Soviet Union. It seems that the temporal evolution of the emissions in the former Soviet Union is better represented in the CMIP6 data set than in the CMIP5 data set, since in the second half of the 20th century the modeled and measured BC deposition fluxes at Akadmii Nauk, Holtedahlfonna and Mt. Elbrus are in much better agreement when using the CMIP6 data.

It is interesting to note that even though Holtedahlfonna and Lomonosovfonna are relatively close to each other (only ca. 100 km apart, at almost same altitude), they have very different BC deposition time series. Lomonosovfonna shows slightly higher deposition fluxes after 1950 with no increase towards the end of the time series, while Holtedahlfonna shows a distinct peak around 1910 and an even stronger peak at the end of the 20th century. These differences can be partly attributed to differences in analytical methods used (SP2 vs. thermo-optical), but may also reflect some local BC source-receptor differences, as well as differences in the state of preservation of the BC signals at the two sites (due to site-specific factors) and/or spatial noise in atmospheric BC deposition, which is presently not well-quantified in Svalbard.

This example illustrates how valuable multiple ice core records are for a comprehensive spatial view, as in some cases local meteorological conditions may affect what particles (free troposphere vs. boundary layer) are recorded in the respective ice core.

**Figures for sensitivity studies of the Inversion:**

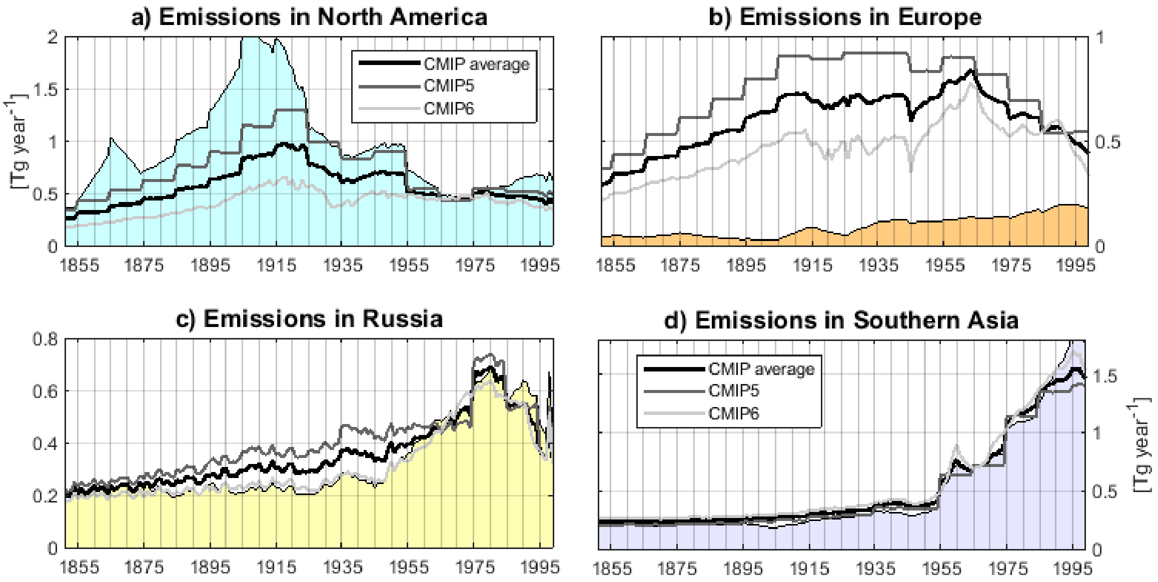


**Fig S9: Same as Fig. 3 but without scaling the modelled concentrations to the measurements obtained in the last decade (1990-2000). The temporal trend for North America, Russia and Asia, stayed similar. However, the European emissions aremuch lower, a consequence mostly of the overestimated BC concentraitons at Colle Gnifetti.**

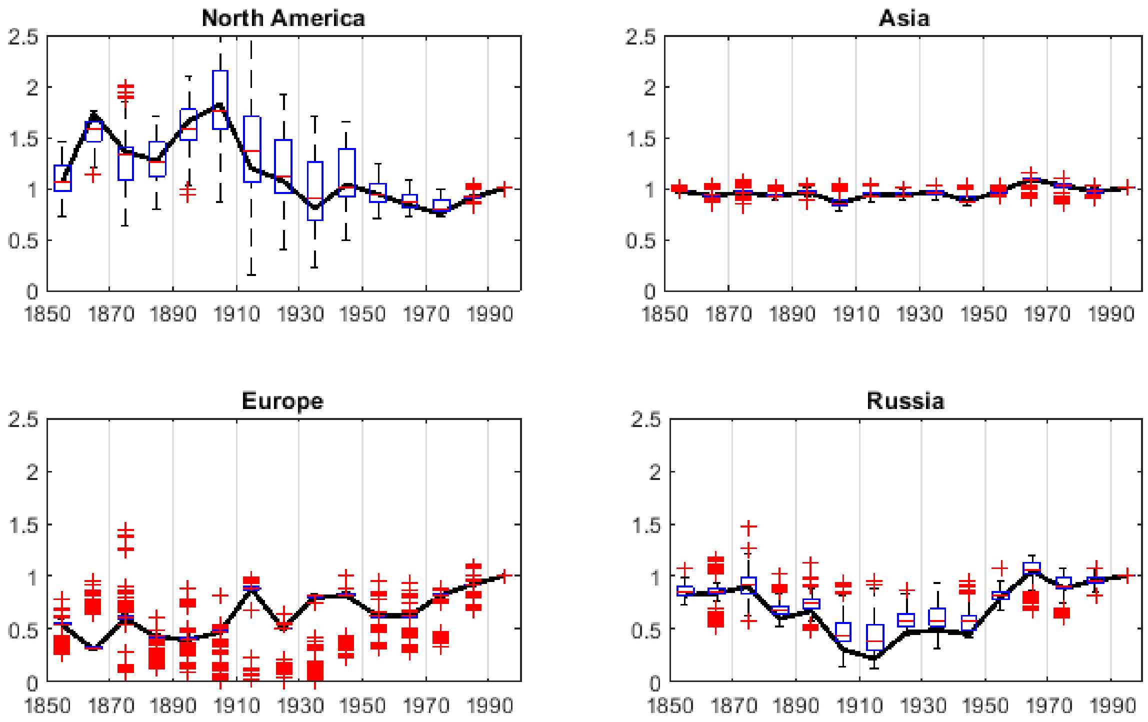


**Fig S10: Sensitivity study: The inversion was calculated as described in the manuscript, but in each iteration 3 of the 13 available ice cores were left out, which gives 364 unique combinations of 11 stations used. The boxes show the 25/75**

**percentile of the set of all possible emission corrections. The black line shows the emission correction used in the manuscript. Russia and North America shows the highest interquartile range, showing that most icecores are sensitive to those emission regions.**

**References SI**


1 Bauer, S. E. *et al.* Historical and future black carbon deposition on the three ice caps: Ice core measurements and model simulations from 1850 to 2100. *Journal of Geophysical Research-Atmospheres* **118**, 7948-7961, doi:10.1002/jgrd.50612 (2013).

2 Beaudon, E. *et al.* Lomonosovfonna and Holtedahlfonna ice cores reveal east west disparities of the Spitsbergen environment since AD 1700. *Journal of Glaciology* **59**, 1069-1083, doi:10.3189/2013JoG12J203 (2013).

3 Grieman, M. M., Aydin, M., McConnell, J. R. & Saltzman, E. S. Burning-derived vanillic acid in an Arctic ice core from Tunu, northeastern Greenland. *Climate of the Past* **14**, 1925-1937, doi:10.5194/cp-14-1625-2018 (2018).

4 Huang, K. *et al.* Russian anthropogenic black carbon: Emission reconstruction and Arctic black carbon simulation. *Journal of Geophysical Research-Atmospheres* **120**, 11306-11333, doi:10.1002/2015jd023358 (2015).

5 Kaspari, S., Skiles, S. M., Delaney, I., Dixon, D. & Painter, T. H. Accelerated glacier melt on Snow Dome, Mount Olympus, Washington, USA, due to deposition of black carbon and mineral dust from wildfire. *Journal of Geophysical Research-Atmospheres* **120**, 2793-2807, doi:10.1002/2014jd022676 (2015).

6 Keegan, K. M., Albert, M. R., McConnell, J. R. & Baker, I. Climate change and forest fires synergistically drive widespread melt events of the Greenland Ice Sheet. *Proceedings of the National Academy of Sciences of the United States of America* **111**, 7964-7967, doi:10.1073/pnas.1405397111 (2014).

7 Klonecki, A. *et al.* Seasonal changes in the transport of pollutants into the Arctic troposphere-model study. *Journal of Geophysical Research-Atmospheres* **108**, 21, doi:10.1029/2002jd002199 (2003).

8 Lim, S. *et al.* Refractory black carbon mass concentrations in snow and ice: method evaluation and inter-comparison with elemental carbon measurement. *Atmospheric Measurement Techniques* **7**, 3307-3324, doi:10.5194/amt-7-3307-2014 (2014).

9 Lim, S. *et al.* Black carbon variability since preindustrial times in the eastern part of Europe reconstructed from Mt. Elbrus, Caucasus, ice cores. *Atmospheric Chemistry and Physics* **17**, 3489-3505, doi:10.5194/acp-17-3489-2017 (2017).

10 McConnell, J. R. & Edwards, R. Coal burning leaves toxic heavy metal legacy in the Arctic. *Proceedings of the National Academy of Sciences of the United States of America* **105**, 12140-12144, doi:10.1073/pnas.0803564105 (2008).

11 McConnell, J. R. *et al.* 20th-century industrial black carbon emissions altered arctic climate forcing. *Science* **317**, 1381-1384, doi:10.1126/science.1144856 (2007).

12 Osmont, D. *et al.* An 800-year high-resolution black carbon ice core record from Lomonosovfonna, Svalbard. *Atmospheric Chemistry and Physics* **18**, 12777-12795, doi:10.5194/acp-18-12777-2018 (2018).

13 Ruppel, M. M. *et al.* Observed and Modeled Black Carbon Deposition and Sources in the Western Russian Arctic 1800-2014. *Environmental Science & Technology* **55**, 4368-4377, doi:10.1021/acs.est.0c07656 (2021).

14 Ruppel, M. M. *et al.* Increase in elemental carbon values between 1970 and 2004 observed in a 300-year ice core from Holtedahlfonna (Svalbard). *Atmospheric Chemistry and Physics* **14**, 11447-11460, doi:10.5194/acp-14-11447-2014 (2014).

15 Schwarz, J. P. *et al.* Assessing Single Particle Soot Photometer and Integrating Sphere/Integrating Sandwich Spectrophotometer measurement techniques for quantifying black carbon concentration in snow. *Atmospheric Measurement Techniques* **5**, 2581-2592, doi:10.5194/amt-5-2581-2012 (2012).

16 Schwarz, J. P. *et al.* Global-scale seasonally resolved black carbon vertical profiles over the Pacific. *Geophysical Research Letters* **40**, 5542-5547, doi:10.1002/2013gl057775 (2013).

17 Sharma, S. *et al.* An evaluation of three methods for measuring black carbon in Alert, Canada. *Atmospheric Chemistry and Physics* **17**, 15225-15243, doi:10.5194/acp-17-15225-2017 (2017).

18 Sigl, M. *et al.* 19th century glacier retreat in the Alps preceded the emergence of industrial black carbon deposition on high-alpine glaciers. *Cryosphere* **12**, 3311-3331, doi:10.5194/tc-12-3311-2018 (2018).

19 Sigl, M. *et al.* A new bipolar ice core record of volcanism from WAIS Divide and NEEM and implications for climate forcing of the last 2000 years. *Journal of Geophysical Research-Atmospheres* **118**, 1151-1169, doi:10.1029/2012jd018603 (2013).

20 Stohl, A. *et al.* Black carbon in the Arctic: the underestimated role of gas flaring and residential combustion emissions. *Atmospheric Chemistry and Physics* **13**, 8833-8855, doi:10.5194/acp-13-8833-2013 (2013).

21 Thomas, J. L. *et al.* Quantifying black carbon deposition over the Greenland ice sheet from forest fires in Canada. *Geophysical Research Letters* **44**, 7965-7974, doi:10.1002/2017gl073701 (2017).

22 van Marle, M. J. E. *et al.* Historic global biomass burning emissions for CMIP6 (BB4CMIP) based on merging satellite observations with proxies and fire models (1750-2015). *Geoscientific Model Development* **10**, 3329-3357, doi:10.5194/gmd-10-3329-2017 (2017).

23 Wendl, I. A. *et al.* Optimized method for black carbon analysis in ice and snow using the Single Particle Soot Photometer. *Atmospheric Measurement Techniques* **7**, 2667-2681, doi:10.5194/amt-7-2667-2014 (2014).

24 Zdanowicz, C. M. *et al.* Historical black carbon deposition in the Canadian High Arctic: a > 250-year long ice-core record from Devon Island. *Atmospheric Chemistry and Physics* **18**, 12345-12361, doi:10.5194/acp-18-12345-2018 (2018).

25 Zennaro, P. *et al.* Fire in ice: two millennia of boreal forest fire history from the Greenland NEEM ice core. *Climate of the Past* **10**, 1905-1924, doi:10.5194/cp-10-1905-2014 (2014).
